# Bacterial shedding and serologic responses following an outbreak of *Salmonella* Typhi in an endemic cohort

**DOI:** 10.1186/s12879-023-08385-8

**Published:** 2023-06-20

**Authors:** Peter I. Johnston, Patrick Bogue, Angeziwa Chunga Chirambo, Maurice Mbewe, Reenesh Prakash, Vanessa Kandoole-Kabwere, Rebecca Lester, Thomas Darton, Stephen Baker, Melita A. Gordon, James E. Meiring

**Affiliations:** 1 Institute of Infection, Veterinary and Ecological Sciences, University of Liverpool, Liverpool, UK; 2 Malawi-Liverpool-Wellcome Trust Clinical Research Programme, Blantyre, Malawi; 3 Department of Medical Laboratory Sciences, Kamuzu University of Health Sciences, Blantyre, Malawi; 4 Department of Infection, Immunity and Cardiovascular Disease, University of Sheffield, South Yorkshire, UK; 5 Cambridge Institute of Therapeutic Immunology and Infectious Disease, University of Cambridge, Cambridge, UK

**Keywords:** Typhoid, *Salmonella* Typhi, Shedding, Sero-surveillance, Outbreak

## Abstract

**Background:**

*Salmonella enterica* serovar Typhi (*Salmonella* Typhi) is the cause of typhoid fever. *Salmonella* Typhi may be transmitted through shedding in the stool, which can continue after recovery from acute illness. Shedding is detected by culturing stool, which is challenging to co-ordinate at scale. We hypothesised that sero-surveillance would direct us to those shedding *Salmonella* Typhi in stool following a typhoid outbreak.

**Methods:**

In 2016 a typhoid outbreak affected one in four residents of a Nursing School in Malosa, Malawi. The Department of Health asked for assistance to identify nursing students that might spread the outbreak to other health facilities. We measured IgG antibody titres against Vi capsular polysaccharide (anti-Vi IgG) and IgM / IgG antibodies against H:d flagellin (anti-H:d) three and six months after the outbreak. We selected participants in the highest and lowest deciles for anti-Vi IgG titre (measured at visit one) and obtained stool for *Salmonella* culture and PCR. All participants reported whether they had experienced fever persisting for three days or more during the outbreak (in keeping with the WHO definitions of ‘suspected typhoid’). We tested for salmonellae in the Nursing School environment.

**Results:**

We obtained 320 paired serum samples from 407 residents. We cultured stool from 25 residents with high anti-Vi IgG titres and 24 residents with low titres. We did not recover *Salmonella* Typhi from stool; four stool samples yielded non-typhoidal salmonellae; one sample produced a positive PCR amplification for a *Salmonella* Typhi target. Median anti-Vi and anti-H:d IgG titres fell among participants who reported persistent fever. There was a smaller fall in anti-H:d IgG titres among participants who did not report persistent fever. Non-typhoidal salmonellae were identified in water sampled at source and from a kitchen tap.

**Conclusion:**

High titres of anti-Vi IgG did not identify culture-confirmed shedding of *Salmonella* Typhi. There was a clear serologic signal of recent typhoid exposure in the cohort, represented by waning IgG antibody titres over time. The presence of non-typhoidal salmonellae in drinking water indicates sub-optimal sanitation. Developing methods to detect and treat shedding remains an important priority to complement typhoid conjugate vaccination in efforts to achieve typhoid elimination.

**Supplementary Information:**

The online version contains supplementary material available at 10.1186/s12879-023-08385-8.

## Background

It is estimated that 1.5 million cases of typhoid fever occurred in Africa (excluding North Africa) in 2017 [1]. Whilst this figure represents a fall in incidence estimates from 1990, localised outbreaks [2–5] map closely to the emergence of genotypes associated with multi-drug resistance (MDR) [6]. Mathematical modelling suggests such outbreaks may be driven by increased transmissibility associated with drug resistant phenotypes [7]. Where *Salmonella enterica* serovar Typhi (*Salmonella* Typhi) is inadequately treated, ongoing shedding is more likely; it is important to detect this in order to break the transmission cycle.

Although stool culture remains the standard test to identify *Salmonella* Typhi shedding, it lacks sensitivity [8]. The relative contribution of temporary shedders to ongoing transmission remains unclear, but is likely to play a larger role in high incidence settings [9, 10].

Serology has been used to complement an outbreak investigation in countries where typhoid is not endemic, resulting in candidate shedders being successfully identified by high anti-Vi antibody titre (anti-Vi IgG) followed by isolation of *Salmonella* Typhi in stool [11, 12]. Anti-Vi antibody titres have been used in attempts to identify carriers in endemic populations (outside of the context of an outbreak), but have led to little recovery of *Salmonella* Typhi [13–15]. Antibody to flagellin (anti-H:d) is a component of the Widal test, and has been proposed as a possible diagnostic marker for typhoid fever [16–19]. Anti-H:d has not previously been evaluated in the context of identifying shedding. To our knowledge, this is the first study to apply these antibody markers to identifying shedding after an outbreak in a country where typhoid is endemic.

In June 2016 an outbreak of febrile illness occurred in Malosa, Malawi. Nine patients presenting to the local hospital had blood culture-confirmed typhoid fever. Cases were traced to a local primary school, secondary school and a residential nursing school. Over the next month the District Health Office identified 101/407 nursing school residents who met the clinical case definition for probable typhoid fever.

Our primary objective was to identify nursing school residents shedding *Salmonella* Typhi after the outbreak, using serology to target subsequent testing of high-risk individuals. We focussed our efforts on the nursing school because students continuing to shed *Salmonella* Typhi might transmit typhoid to other healthcare institutions when they dispersed for work placements. Secondary objectives were to identify any environmental source of *Salmonella* Typhi within the nursing school and to explore serologic responses to typhoid exposure, as determined by self-reported, persistent fever.

## Methods

We performed a prospective observational study comprising two study visits, conducted three and six months after the Malosa outbreak.

Any person over 18 years of age who had been resident in the nursing school between 1st June 2016 and 31st August 2016 was eligible to participate. We enrolled participants and conducted study visit one simultaneously in October 2016, three months after the outbreak.

We collected a blood sample from each participant and completed a structured case record. This included self-report of whether the participant had a persistent fever (defined as lasting three or more days) during the outbreak. Those who reported persistent fever were considered to have been “suspected typhoid” cases based on the WHO case finding definition [20].

We identified participants whose three-month anti-Vi IgG levels were in the top 10% of the cohort and participants whose anti-Vi IgG were in the lowest 10% of the cohort. These participants were approached and asked to provide a stool sample. Samples were collected between the three- and six-month study visits, after anti-Vi IgG results became available. Stool was sent for culture and polymerase chain reaction (PCR) testing.

The cohort was approached again in January 2017, six months after the outbreak. A further blood sample was obtained for repeat serology.

### Laboratory procedures

We determined anti-Vi IgG antibody concentration using a commercial Enzyme-linked immunosorbent assay (ELISA) in accordance with manufacturer’s instructions (VaccZyme™ The Binding Site, Birmingham, UK). We derived antibody concentration in ELISA units/ml (EU/ml) from the optical density using a standardised curve-fitting logistic method.

Anti-H:d IgM and IgG were performed at the Oxford University Clinical Research Unit in Vietnam, using in-house ELISAs, as previously described [21].

Stool samples were collected in sterile sample pots and transferred to the laboratory on the same day. Stool was pre-enriched in Selenite broth at 37˚C for 18–24 h before being sub-cultured on xylose lysine deoxyxholate (XLD) agar. Single colonies from XLD were sub-cultured on MacConkey and sheep blood agar (SBA) plates. Colonies from SBA plates were used for biochemical tests. Analytical Profile Index (API 20E) tests were used for *Salmonella* identification. Following the White-Kauffmann-Le Minor scheme, serotyping was done using polyvalent O and H, O4, O9, Hd, Hg, Hi, Hm, and Vi antisera (Pro-Lab Diagnostics).

PCR testing was used alongside routine culture to increase the yield from stool. DNA was extracted from Selenite broth pre-enriched stool specimens using QIAamp Fast DNA Stool Mini Kit (Qiagen). A pan *Salmonella* PCR targeting the *ttr* gene, encoding tetrathionate reductase, and a multiplex PCR with a pan-*Salmonella* invasion A gene, *S.* Typhi fimbriae gene, and the kit’s internal control were performed [22, 23].

### Environmental sampling

Malosa Dam provides water for the nursing school and secondary school via a gravity fed water pipe feeding in to a holding tank. The water is reportedly chlorinated daily after leaving the holding tank.

We took 36 environmental samples, in-duplicate, over two days. These comprised stool from food-handlers, food and tap water from the kitchens and water from the dam. Samples were cultured and sero-typed as described above.

### Statistical methods

We treated serologic data as a metric continuous variable, and our surrogate measure of typhoid exposure as dichotomous (fever for ≥ 3 days or not).

We tested continuous variables for normality using the Shapiro-Wilks test before and after log-transformation. We used nonparametric tests where data were not normally distributed.

We used paired significance tests to compare change in antibody titre within the population as a whole and change in antibody titre depending upon whether the case definition for suspected typhoid had been met. We used unpaired significance tests to compare antibody titres between participants who did and did not meet the case definition for suspected typhoid at each study visit. Only participants who submitted serum at three and six months contributed to paired analyses.

Statistical analyses were carried out using the free to use, open source statistical package ‘R’ version 4.1.1 [24]. The significance threshold was set at 0.05 for all tests.

### Ethical approval and consent to participate

All participants were adults aged 18 years or more. We ensured that participant information materials were available in English and Chichewa languages. We obtained written informed consent from each enrolled participant, which included consent for the described laboratory analyses. Our protocol stated that participants who were stool culture positive for *Salmonella* Typhi would be advised to take either 28 days of ciprofloxacin or 14 days of azithromycin with the aim of eradicating carriage and preventing onward transmission, but this proved not to be necessary. We conducted study procedures in accordance with a protocol approved by the Malawi College of Medicine Research Ethics Committee, P.10/16/2043.

## Results

We recruited 374 participants from a total population of 407 nursing school residents. 368 participants submitted blood samples three months post outbreak. 320 participants submitted blood at three months and six months. We obtained stool samples from 25 to 36 participants whose anti-Vi IgG response was in the highest decile, and from 24 to 36 participants whose anti-Vi IgG response was in the lowest decile. The characteristics of included participants are shown in Table 1.

**Table 1:** Baseline characteristics of participants, self-reported symptoms in those with and without suspected typhoid, and antibody titres amongst those with and without suspected typhoid at visits one and two

Meets ‘suspected typhoid’ case definition	Does not meet ‘suspected typhoid’ case definition	Test of difference			
Participants (N = 368)	*156*	*212*		NA	
Age in years (mean) *(range)* *(SD)*	*22.6* *18–54* *4.4*	*24.2* *18–42* *6.3*		*p = 0.09 (unpaired t-test)*	
gender (proportion female, (%))	*78/156 (50)*	*106/212 (50)*		*p = 1 (chi-squared test)*	
Treated with antibiotics (%)	*140/156 (90)*	*16/212 (7.5)*		*p = < 0.001 (chi-squared test)*	
Median anti-Vi IgG titre at visit one (EU/ml)	*8.08 (IQR 17.9)*	*3.7 (IQR 12.4)*		*p = 0.11 (Mann-Whitney-U test)*	
Median anti-Vi IgG titre at visit two (EU/ml)	*3.7 (IQR 17.7)*	*3.7 (IQR14.4)*		*p = 0.86 (Mann-Whitney-U test)*	
Median anti-H:d IgM antibody titre at visit one (EU/ml)	*55.2 (IQR 51.4)*	*52.1 (IQR 55.9)*		*p = 0.34 (Mann-Whitney-U test)*	
Median anti-H:d IgM antibody titre at visit two (EU/ml)	*54.2 (IQR 44.9)*	*55.3 (IQR 56.1)*		*p = 0.92 (Mann-Whitney-U test)*	
Median anti-H:d IgG antibody titre at visit one (EU/ml)	*87.8 (IQR 85.1)*	*82.4 (IQR 88.4)*		*p = 0.27 (Mann-Whitney-U test)*	
Median anti-H:d IgG antibody titre at visit two (EU/ml)	*77.4 (IQR 57)*	*79.2 (IQR 65.7)*		*p = 0.80 (Mann-Whitney-U test)*	
Headache, N (%)	*139/156 (89)*	*39/212 (18.4)*		*p = < 0.001 (chi-squared test)*	
Abdominal pain, N (%)	*82/156 (52.6)*	*25/212 (11.8)*		*p = < 0.001 (chi-squared test)*	
Diarrhoea, N (%)	*62/156 (39.7)*	*29/212 (13.7)*		*p = < 0.001 (chi-squared test)*	
Constipation, N (%)	*31/156 (19.9)*	*10/212 (4.7)*		*p = < 0.001 (chi-squared test)*	
Nausea, N (%)	*60/156 (38.5)*	*13/212 (6.1)*		*p = < 0.001 (chi-squared test)*	

### Stool culture

**Fig. 1:**
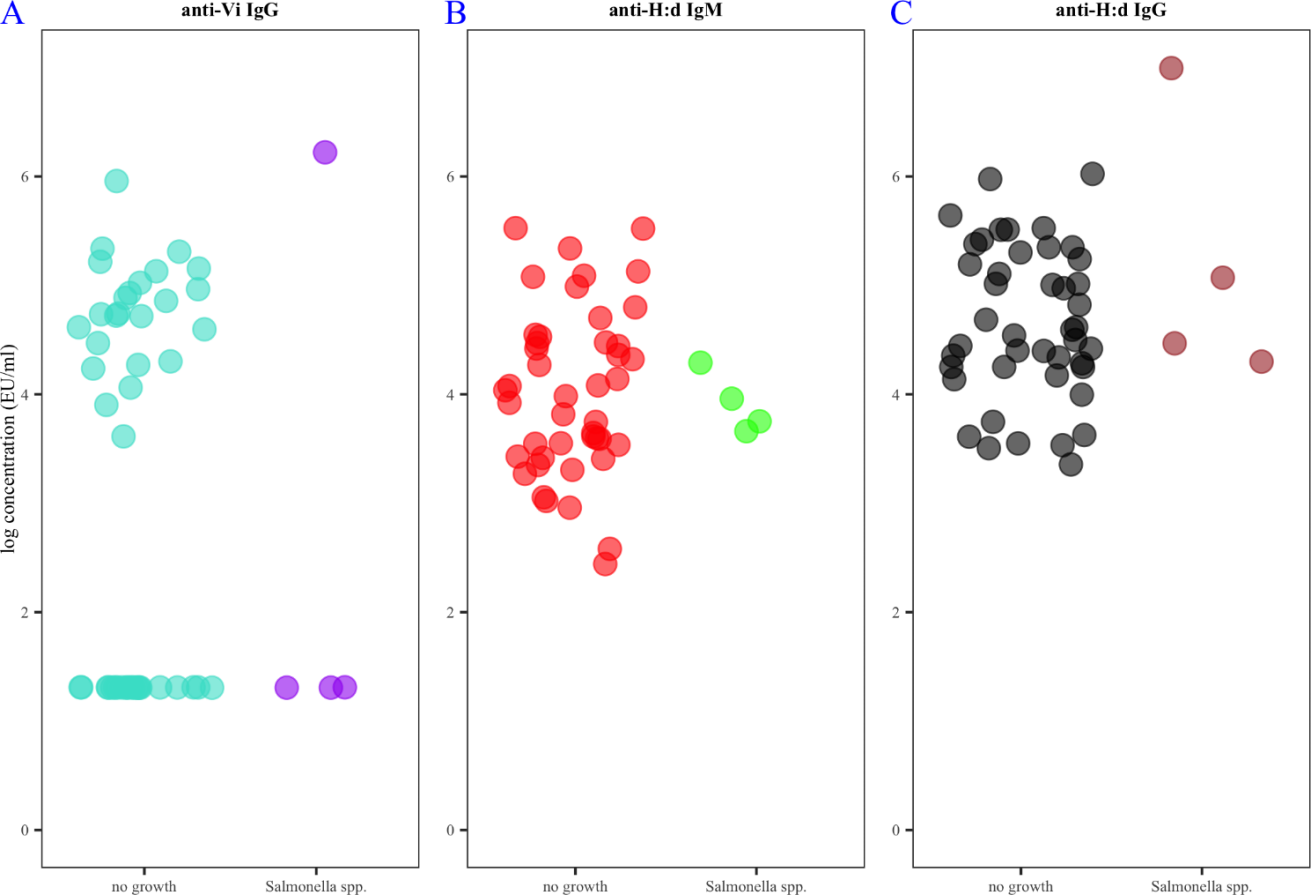
**A.** Log anti-Vi IgG concentration at the three month visit among participants from whom Salmonella spp. was cultured (purple) compared to culture-negative participants(turquoise). **B.** log anti-H:d IgM concentration at the three month visit among participants from whom Salmonella spp. was cultured (green) compared to culture-negative participants(red). **C.** log anti-H:d IgG concentration at the three month visit among participants from whom Salmonella spp. was cultured (brown) compared to culture-negative participants (black)

Of 49 participants who submitted stool for culture, none were culture positive for *Salmonella* Typhi in stool. Four participants had *Salmonella* spp. in stool on routine culture, and all four of these samples produced a positive amplification of the *ttr* PCR target. Although we performed serotyping according to the Kauffman-Le minor scheme, the eventual classification recorded was that these were non-typhoidal salmonellae – the serotype data is not available. Three of these participants were in the lowest decile for anti-Vi IgG level at visit one, one was in the highest decile (Fig. 1).We did not recover viable *Salmonella* Typhi from any of the stool samples collected. One stool sample produced a positive amplification of the *Salmonella* Typhi fimbriae gene target on multiplex PCR. The participant that produced this sample had the highest three-month anti-Vi IgG and anti-H:d IgG titres among participants submitting stool (Supplemental Fig. 1).

### Antibody responses

**Fig. 2:**
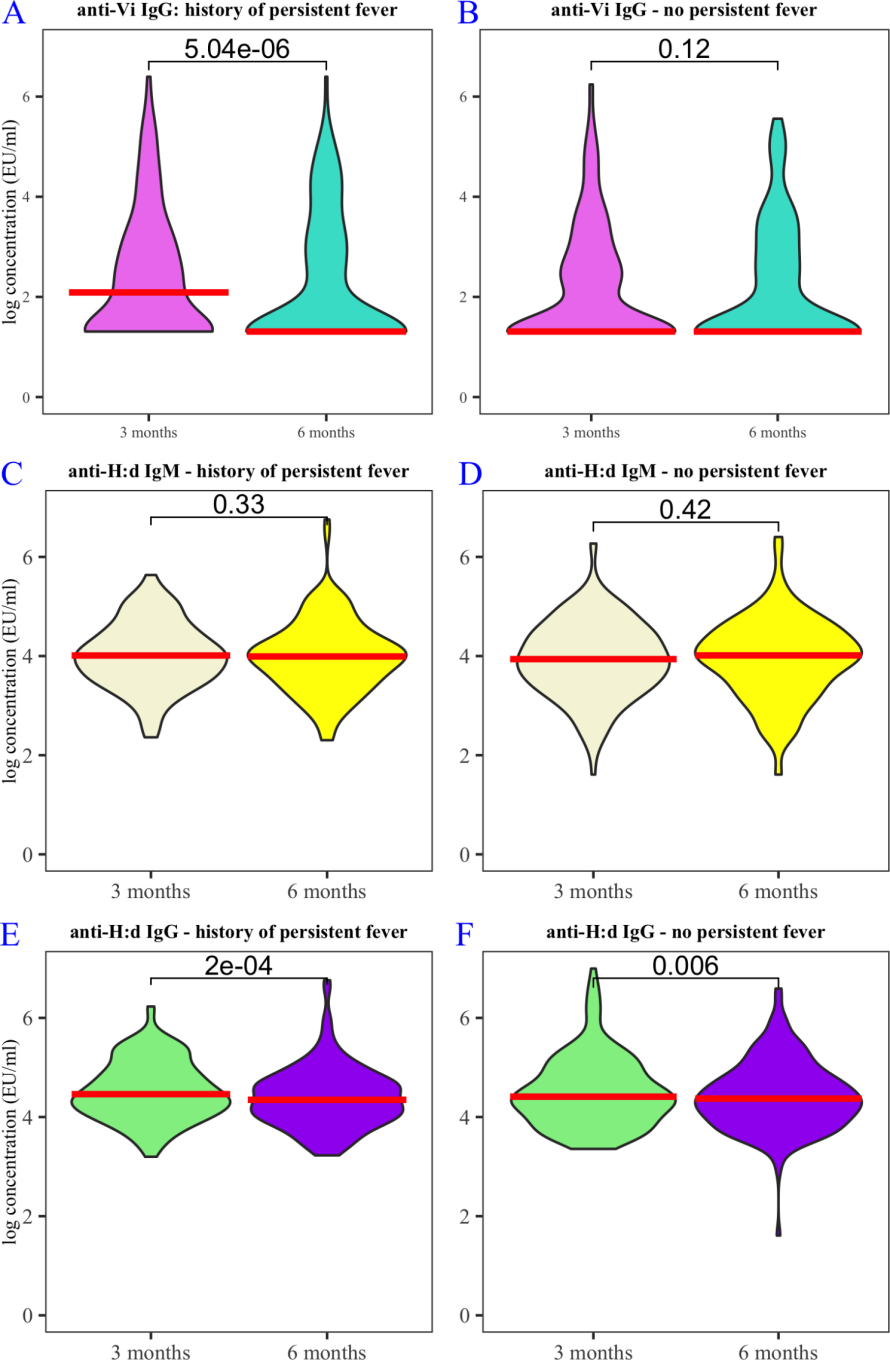
**A.** log anti-Vi IgG concentration amongst participants with persistent fever, comparing titres at 3 months (lilac) and 6 months (turquoise) post-outbreak. **B.** log anti-Vi IgG concentration amongst participants without persistent fever, comparing titres at 3 months (lilac) and 6 months (turquoise) post-outbreak. **C.** log anti-H:d IgM concentration amongst participants with persistent fever, comparing titres at 3 months (beige) and 6 months (yellow) post-outbreak. **D.** log anti-H:d IgM concentration amongst participants without persistent fever, comparing titres at 3 months (beige) and 6 months (yellow) post outbreak. **E.** log anti-H:d IgG concentration amongst participants without persistent fever, comparing titres at 3 months (green) and 6 months (purple) post-outbreak. **F.** log anti-H:d IgM concentration amongst participants without persistent fever, comparing titres at 3 months (green) and 6 months (purple) post-outbreak. All graphs: red crossbar shows median antibody concentration; brackets show p-values (Wilcoxon signed rank test)

Among participants reporting persistent fever there was a significant fall in anti-Vi IgG between three and six months (median titre 8.08 to 3.7 EU/ml (*p = < 0.001, Wilcoxon signed rank test*). This was not true of participants who did not report persistent fever (median titre 3.7 to 3.7 EU/ml, *p = 0.12, Wilcoxon signed rank test*) (Fig. 2.)

Among participants with persistent fever there was a significant fall in anti-H:d IgG between three and six months (median titre 87.8 to 77.4 EU/ml (*p = < 0.001, Wilcoxon signed rank test*). Among participants without persistent fever, there was also a significant fall in anti-H:d IgG between three and six months (median titre 82.4 to 79.2 EU/ml, *p = 0.006, Wilcoxon signed rank test*) (Fig. 2.)

There was no significant change in anti-H:d IgM between three and six months, either among those with persistent fever (median titre 55.2 to 54.2 EU/ml (*p = 0.33, Wilcoxon signed rank test*) or without persistent fever (median titre 52.1 to 55.3 EU/ml (*p = 0.42, Wilcoxon signed rank test*). (Fig. 2.)

### Environmental sampling

Non-typhoidal salmonellae were identified from tap water in the kitchen of the nursing school and in water from Malosa dam. No *S.* Typhi was identified.

## Discussion

Our study shows that, even in a population where there had been a recent outbreak of typhoid fever, sero-surveillance could not identify culture-confirmed shedding of *Salmonella* Typhi. Sero-surveillance combined with PCR may have identified one participant shedding *Salmonella* Typhi. Anti-Vi IgG titres fell amongst participants reporting persistent fever during the outbreak. Anti-H:d IgG titres fell amongst participants with and without persistent fever. There was no change in anti-H:d IgM, irrespective of fever status. We did not find an environmental reservoir of *Salmonella* Typhi, but non-typhoidal salmonellae were present in water samples from the reservoir supplying the nursing school, and a tap in the kitchen. The presence of these bacteria indicate contamination and under-treatment of the water supply, and cast suspicion that this could have been a route through which the outbreak was sustained. Feedback from this investigation has resulted in more robust chlorination procedures.

Our study population was young (mean 23.5 years). Although we did not formally ascertain this, biliary pathology is rare in sub-Saharan Africa [25]. A similar study design might have detected *Salmonella* Typhi shedding amongst an older cohort, or in an endemic setting where rates of biliary disease are high. 140 of 156 participants meeting the case definition for ‘suspected typhoid’ received antibiotics, compared with 16 of 212 not meeting the case definition – it is likely that antibiotic administration reduced the amount of *Salmonella* Typhi that could be recovered post-outbreak. Our study design was contingent upon how swiftly ethical approval could be obtained after the outbreak. Thus, we selected three months for the first study visit, given that the immediate serologic signal caused by recent enteric fever would have started to settle, enabling us to identify participants with persistently elevated antibody titres attributable to ongoing shedding. It may be that we would have identified acute temporary shedding (shedding occurring within three months of illness)[10] if we had taken stool samples at an earlier time-point, but this would not have allowed us to test our hypothesis that presently elevated antibody titre was associated with ongoing shedding. Unfortunately, the serotypes of the non-typhoidal salmonellae we recovered from stool and from water were not recorded, and we can only state that they were non-typhoidal salmonellae (*Salmonella enterica* subspecies *enterica*).

Participants from the Nursing College were on clinical placement in multiple institutions across Southern Malawi. This led to participants being lost to follow up between 3 and six months. Given the intermittent nature of shedding, several samples over time would have increased our chances of detecting *Salmonella* Typhi by culture. Another approach (had resource allowed) would have been to take a stool sample from all participants, regardless of antibody titre, and thence investigate the difference in titres between culture confirmed shedders with that of the rest of the cohort. One participant was identified as having PCR amplification of the *fimbriae* target. This suggests that they may have been carrying / shedding *Salmonella* Typhi, but this could not be verified through culture confirmation. This participant had the highest anti-Vi IgG and anti-H:d IgG titres of all those who submitted stool.

Although we have no pre-outbreak serologic data to base this on, antibody titres to our selected antigens are likely to have risen and peaked around six weeks after the outbreak [26]. As described by Voysey and Pollard, decreases in antibody concentrations provide serologic evidence of infection [27]. This is supported by the fall in anti-Vi IgG (amongst those with suspected typhoid) and the cohort-wide fall in anti-H:d IgG between three and six months. Anti-H:d IgG has been shown to fall swiftly after controlled human infection, and this may explain why the signal was observed across the cohort for this antigen but only in those with suspected typhoid for the Vi antigen [19]. The static anti-H:d IgM concentration suggests that any change in titre precipitated by the outbreak has been and gone at three months.

The Vi (virulence) antigen comprises a polysaccharide expressed on the surface of *Salmonella* Typhi, as well as *Salmonella* Paratyphi C, Citrobacter *freundii* and *Salmonella* Dublin [28, 29]. IgG antibodies to the Vi antigen have been detected in high titres among microbiologically confirmed shedders of *Salmonella* Typhi [13, 30, 31]. *Salmonella* Typhi shedders have been identified through their high anti-Vi titre in the context of two outbreaks in the United States [11, 12]. Yet the role of anti-Vi IgG for population screening for Typhoid shedding remains unclear: a 2004 study obtained sera from 3209 adults in a Typhoid-endemic area (Vietnam). Multiple rectal swabs were taken from 103 participants with the highest anti-Vi IgG titres at different time points: no *Salmonella* Typhi was isolated [14]. Although we obtained fewer faecal specimens, our study population had known recent exposure to typhoid. The paucity of *Salmonella* Typhi detection in both settings suggests that anti-Vi IgG might be elevated for reasons other than biliary carriage, such as cross-reactivity with other bacteria that express the Vi antigen, or convalescence from acute typhoid fever. It is plausible that both factors would be more significant in areas of the world where typhoid fever is endemic, explaining the geographical discrepancy. A Chilean study comparing anti-Vi IgG amongst bacteriologically proven *Salmonella* Typhi carriers and healthy control subjects and those with acute typhoid fever supports our finding that anti-Vi IgG is a non-specific marker in an endemic setting: although the authors demonstrated higher antibody titre among the shedders, one quarter of chronic carriers did not have a high anti-Vi IgG titre [13]. There was no licensed typhoid vaccine in Malawi at the time that our study was conducted. We are therefore confident that our cohort’s serologic responses have not been impacted by prior immunisation.

Flagellin proteins comprise the bacterial flagellum, which is used for motility – predominantly by Gram-negative bacteria [32]. Flagellin epitopes are distinct among *Salmonella* serovars, which is why they may be used in serotyping [33]. Elevated anti-H IgG titres have been found amongst people infected by other *Salmonella spp.*, other Enterobacteriaceae, and even *Staphylococcus aureus* [34, 35]. In our study, we show elevated titres of anti-H:d IgG amongst participants with and without persistent fever (although the fall between visit one and two was statistically greater amongst those with persistent fever). In our study setting, it is likely that participants will have been exposed to other Enterobacteraciae and *Salmonella spp.*, which may explain why we observed elevated anti-H:d titres across the cohort. This illustrates the difficulty of using serology to identify shedding in endemic settings – a combination of antigens may be more specific.

A recent study utilising culture confirmed carriers who underwent cholecystectomy in Nepal found 13 putative antigens in sera that were expressed highly in the carrier state, and which might bear closer scrutiny in future studies looking for shedding [36]. Future studies should focus on robust microbiological confirmation of *Salmonella* Typhi shedding status to provide clear comparators for prospective serologic markers. The detection of carriers will require repeated stool sampling over time, and newer PCR techniques may increase sensitivity over culture alone [22, 37].

Despite the success of conjugate typhoid vaccines in preventing typhoid cases, indirect protection is yet to be demonstrated [38]. Wider public health measures, including identifying those who continue to shed *Salmonella* Typhi after infection, remain key to reducing transmission and to ultimately eradicating typhoid.

## Electronic supplementary material

Below is the link to the electronic supplementary material.

**Supplemental Figure 1 A**. Log anti-Vi IgG concentration at the three month visit among participants who did and did not amplify the fimbriae PCR target from stool. Green coloured dots represent participants who were culture-negative for Salmonella spp. from stool, turquoise coloured dots represent those who were culture-positive for Salmonella spp from stool. **B**. log anti-H:d IgM at the three month visit among participants who did and did not amplify the fimbriae PCR target from stool. Yellow coloured dots represent participants who were culture-negative for Salmonella spp. from stool, purple coloured dots represent those who were culture-positive for Salmonella spp from stool. **C**. log anti-H:d IgG at the three month visit among participants who did and did not amplify the fimbriae PCR target from stool. Orange coloured dots represent participants who were culture-negative for Salmonella spp. from stool, grey coloured dots represent those who were culture-positive for Salmonella spp from stool.

## Data Availability

The datasets used and/or analysed during the current study are available from the corresponding author on reasonable request.
