# Supplementary material for: Bacterial shedding and serologic responses following an outbreak of Salmonella Typhi in an endemic cohort
Source: BMC Infect Dis. 2023 Jun 20;23:416. doi: 10.1186/s12879-023-08385-8 (PMC10280929; doi:10.1186/s12879-023-08385-8)
Supplement: Supplementary file 1 — Supplemental Figure 1 A. Log anti-Vi IgG concentration at the three month visit among participants who did and did not amplify the fimbriae PCR target from stool. Green coloured dots represent participants who were culture-negative for Salmonella spp. from stool, turquoise coloured dots represent those who were culture-positive for Salmonella spp from stool. B. log anti-H:d IgM at the three month visit among participants who did and did not amplify the fimbriae PCR target from stool. Yellow coloured dots represent participants who were culture-negative for Salmonella spp. from stool, purple coloured dots represent those who were culture-positive for Salmonella spp from stool. C. log anti-H:d IgG at the three month visit among participants who did and did not amplify the fimbriae PCR target from stool. Orange coloured dots represent participants who were culture-negative for Salmonella spp. from stool, grey coloured dots represent those who were culture-positive for Salmonella spp from stool. [file 12879_2023_8385_MOESM1_ESM.docx]

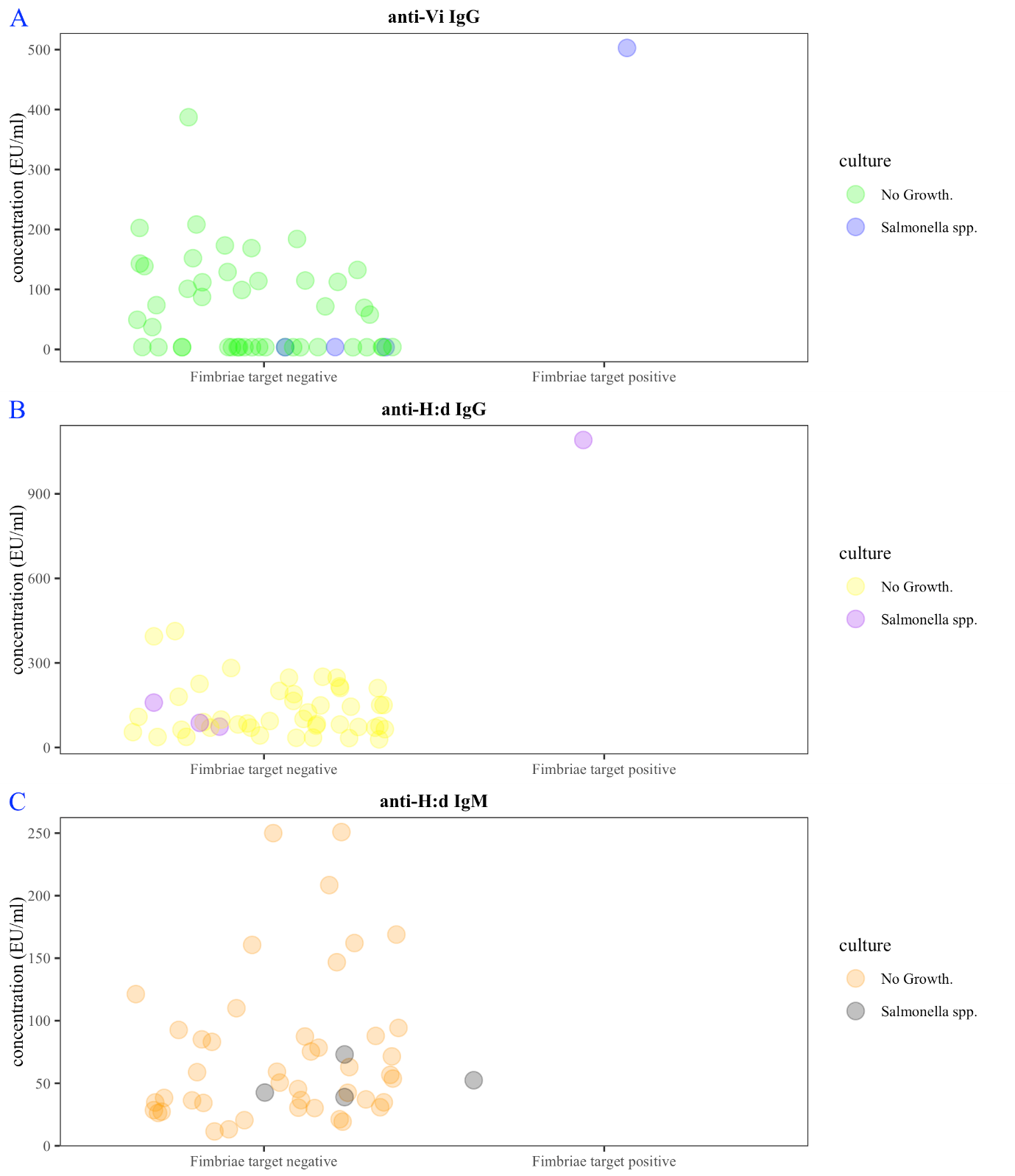


Supplemental Figure 1 **A.** Log anti-Vi IgG concentration at the three month visit among participants who did and did not amplify the fimbriae PCR target from stool. Green coloured dots represent participants who were culture-negative for Salmonella spp. from stool, turquoise coloured dots represent those who were culture-positive for Salmonella spp from stool. **B.**  log anti-H:d IgM at the three month visit among participants who did and did not amplify the fimbriae PCR target from stool. Yellow coloured dots represent participants who were culture-negative for Salmonella spp. from stool, purple coloured dots represent those who were culture-positive for Salmonella spp from stool. **C.** log anti-H:d IgG at the three month visit among participants who did and did not amplify the fimbriae PCR target from stool. Orange coloured dots represent participants who were culture-negative for Salmonella spp. from stool, grey coloured dots represent those who were culture-positive for Salmonella spp from stool.
